# Association between height loss and mortality in the general population

**DOI:** 10.1038/s41598-023-30835-1

**Published:** 2023-03-03

**Authors:** Tsuyoshi Iwasaki, Hiroshi Kimura, Kenichi Tanaka, Koichi Asahi, Kunitoshi Iseki, Toshiki Moriyama, Kunihiro Yamagata, Kazuhiko Tsuruya, Shouichi Fujimoto, Ichiei Narita, Tsuneo Konta, Masahide Kondo, Masato Kasahara, Yugo Shibagaki, Tsuyoshi Watanabe, Junichiro J. Kazama

**Affiliations:** 1 Department of Nephrology and Hypertension, Fukushima Medical University, 1 Hikariga-oka, Fukushima, Fukushima 960-1295 Japan; 2 Steering Committee of The Japan Specific Health Checkups (J-SHC) Study Group, Fukushima, Japan

**Keywords:** Predictive markers, Epidemiology, Risk factors

## Abstract

Height loss is caused by osteoporosis, vertebral fractures, disc reduction, postural changes, and kyphosis. Marked long-term height loss is reportedly associated with cardiovascular disease and mortality in the elderly. The present study investigated the relationship between short-term height loss and the risk of mortality using the longitudinal cohort data of the Japan Specific Health Checkup Study (J-SHC). Included individuals were aged 40 years or older and received periodic health checkups in 2008 and 2010. The exposure of interest was height loss over the 2 years, and the outcome was all-cause mortality over subsequent follow up. Cox proportional hazard models were used to examine the association between height loss and all-cause mortality. Of the 222,392 individuals (88,285 men, 134,107 women) included in this study, 1436 died during the observation period (mean 4.8 ± 1.1 years). The subjects were divided into two groups based on a cut-off value of height loss of 0.5 cm over 2 years. The adjusted hazard ratio (95% confidence interval) was 1.26 (1.13–1.41) for exposure to height loss ≥ 0.5 cm compared to height loss < 0.5 cm. Height loss ≥ 0.5 cm correlated significantly with an increased risk of mortality compared to height loss < 0.5 cm in both men and women. Even a small decrease in height over 2 years was associated with the risk of all-cause mortality and might be a helpful marker for stratifying mortality risk.

## Introduction

Height loss, which is caused by disc reduction^1^, change in posture^2^, and vertebral fractures^3^, is known to occur in the long term beginning in the fourth decade of life and accelerating in older age (70 s and above). Although osteoporosis is thought to be one of the main factors associated with height loss, especially in the elderly, the resultant height loss affects the normal functioning of the cardiopulmonary and gastrointestinal systems^4,5^, which might cause malnutrition and decrease in skeletal muscle mass (sarcopenia)^6^. Several previous studies involving observation for several to a dozen years have reported that marked height loss in the long term is associated with mortality^7–11^, fractures^8,12^, and cardiovascular diseases^9–11,13^ in the elderly. In these previous studies, relationships between height loss and mortality risk were reported separately in men^11^ and women^8,9^. Auyeung et al. reported that height loss of over 2 cm in 4 years was related to an increased risk of all-cause mortality only in men^7^, suggesting a sex difference in these relationships. Despite these reports, trends in height change have received little clinical attention as an indicator of health status, other than as a marker of osteoporosis^14–16^. Furthermore, the height loss related to an increased risk of mortality in the previous studies was reportedly 2–5 cm^7–11^, and the impact of smaller decreases in height in the short term on mortality has not been elucidated. Hypothesizing that even a smaller height loss could be related to mortality risk, we investigated the association between short-term height loss over 2 years and mortality risk in a nationwide Japanese population using the longitudinal cohort data of the Japan Specific Health Checkup Study (J-SHC).

## Materials and methods

### Study population

In 2008, the Japanese government launched the National Health Examination Program to prevent lifestyle-related diseases and aid in the early diagnosis and intervention for metabolic syndrome. Clinical details of the Japan Specific Health Checkup Study (J-SHC) have been described previously^17–19^. Using the data from the J-SHC, we obtained data from seven prefectures (Fukushima, Ibaraki, Osaka, Fukuoka, Miyazaki, Okinawa and Niigata). The review committees of each research institution provided ethical approval for this study. Included individuals were aged 40 years or older and received periodic health check-ups in 2008 and 2010. We excluded participants who had missing data, and whose height changed by ≥ 5 cm in 2 years, considering it a measurement error.

All procedures in this study were performed in accordance with the Declaration of Helsinki and the Ethical Guidelines for Epidemiological Studies published by the Ministry of Education, Science and Culture and the Ministry of Health, Labour and Welfare of Japan. The requirement for informed consent was waived because the data are anonymous. The Ethics Committee of Fukushima Medical University approved the research protocol (#1485 and #2771) and waived the need for informed consent for the requirement because the data were anonymous.

### Measurement and definition

Participants visited a pre-designated clinic or hospital and answered questionnaires regarding history of stroke, heart disease and kidney disease, and lifestyle habits such as smoking, diet, and alcohol consumption. Physicians involved in the study conducted a physical examination of each participant and reviewed their medical history to ensure accurate information. Trained staff then measured height, weight, blood pressure and waist circumference. Height was measured to the nearest 0.1 cm using a stadiometer, with the participants standing upright without shoes. We calculated body mass index by dividing weight (in kilograms) by the square of the height (in meters). Blood pressure was measured in the sitting position using a standard or automatic sphygmomanometer after resting for 5 min. Blood samples were collected from all participants after an overnight fast.

Hypertension was defined as a blood pressure of ≥ 140/90 mmHg or on antihypertensive medication. Diabetes mellitus was defined in accordance with American Diabetes Association Guidelines, and was identified by a fasting plasma glucose concentration ≥ 126 mg/dL, glycated hemoglobin (HbA1c) value ≥ 6.5%, or the use of antidiabetic medication. HbA1c was estimated using the National Glycohemoglobin Standardization Program (NGSP) equivalent value calculated by the following equation: HbA1c (NGSP) = HbA1c (Japan Diabetes Society) + 0.4%^20^. Dyslipidemia was defined as triglycerides ≥ 150 mg/dL, high-density lipoprotein (HDL) cholesterol ≤ 40 mg/dL, low-density lipoprotein (LDL) cholesterol ≥ 140 mg/dL, or on therapeutic medication^21^. We considered stroke and cardiovascular diseases to be present in participants who reported being diagnosed or treated for stroke or cardiovascular diseases, respectively, in the questionnaire.

### Exposure and outcomes

The exposure of interest in this study was height loss over 2 years. Height loss was calculated as baseline (2008) height minus height at follow-up (2010), with a positive value indicating height loss. Next, we divided the participants into two groups based on cut-off values of height loss of 0.5 cm, 1.0 cm, or 1.5 cm over 2 years. The primary outcome was all-cause mortality, and the secondary outcome was cardiovascular mortality during follow-up until the end of the study period (1 April 2015). We verified the date and cause of death in the death certificate database with permission from the Ministry of Health, Labour and Welfare. The cause of death was coded according to the International Classification of Diseases, 10th revision (ICD-10). Cardiovascular mortality was defined as mortality in which the cause of death corresponded to the following ICD-10 codes: I20-51, I60-77, I99.

### Statistical analyses

The variables are presented as median ± standard deviation, medians with interquartile (IQR) ranges, or frequencies (proportions), as appropriate. All analyses were conducted in the entire cohort, as well as separately for men and women. Differences in baseline characteristics between categories were assessed using a t-test, Mann–Whitney test, or chi-squared test. The Cox proportional hazard model was used to examine the association between height loss and all-cause mortality and cardiovascular mortality. Schoenfeld residuals were used to test proportional hazards assumptions. For each analysis, the following adjustment models were applied: (1) Model 1, which included age, sex and baseline height; and (2) Model 2, which included all the covariates in Model 1 plus body mass index, hypertension, diabetes, dyslipidemia, history of stroke, history of cardiovascular disease and current smoking.

For sensitivity analysis, the relationship between height loss and mortality was estimated using restricted cubic spline functions with four knots at the 5th, 35th, 65th, and 95th percentiles of each index. The restricted cubic spline method evaluates the relationship between the response variable and a vector of covariates. This method can help prevent the problems resulting from inappropriate linearity assumptions, and is widely used to assess non-linear associations^22–24^. We used the multiple imputation method with 20 datasets in all regression analyses. A P value of less than 0.05 was considered to be significant. All analyses were conducted using STATA MP, version 15.1 (Stata Corp, College Station, TX, USA).

## Results

### Participants’ characteristics

**Figure 1:**
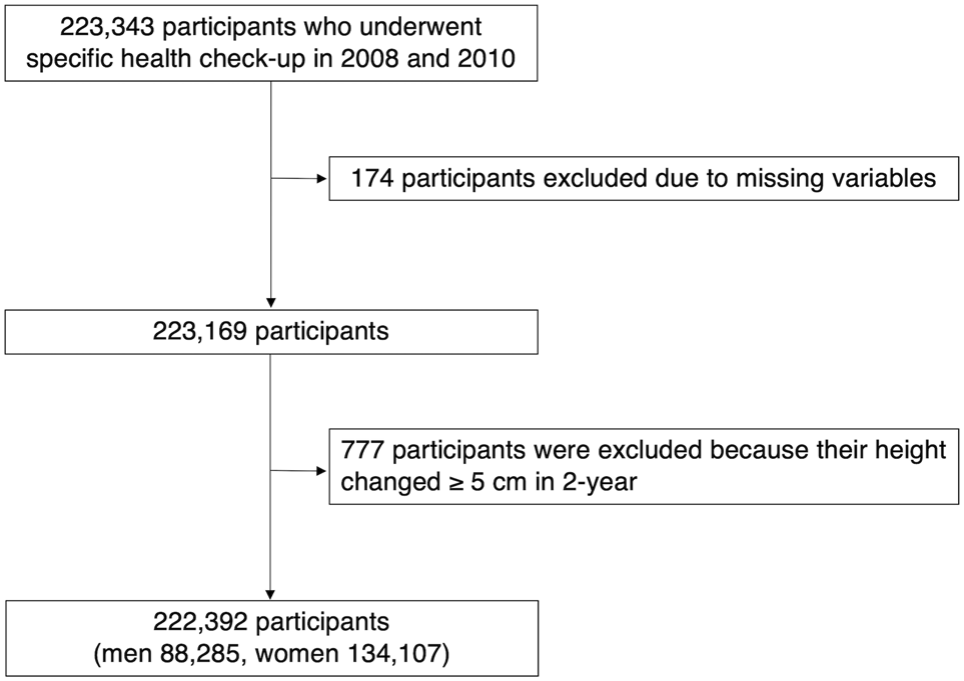
Flow chart for selection of the study participants.

A total of 222,392 individuals (88,285 men, 134,107 women) were included in this study (Fig. 1). We evaluated the baseline characteristics of all the participants (Table 1), and of men (Table 2) and women (Table 3) separately. The mean age of all participants at baseline was 63.4 ± 7.3 years. Using different cut-off points for height loss, the proportions of patients with height loss greater than 0.5 cm, 1.0 cm, 1.5 cm, and 2.0 cm over 2 years were 31.2%, 10.6%, 3.5%, and 1.5%, respectively. Participants in the height loss ≥ 0.5 cm group were older and included more women. Height loss was significantly greater in women than men (0.3 ± 0.7 vs. 0.2 ± 0.6 cm, *P* < 0.001). Among men, participants with height loss ≥ 0.5 cm were older, taller and had lower body weight at baseline. As for comorbidities, prevalence of hypertension and cardiovascular disease history were higher in men with height loss ≥ 0.5 cm. In women, participants with height loss ≥ 0.5 cm weighed more and had higher blood pressure at baseline.

**Table 1:** Baseline (2008) characteristics of all included participants stratified into 2-year height loss < 0.5 cm and ≥ 0.5 cm.

	Total	Height loss		P value
		< 0.5 cm	≥ 0.5 cm	
	n = 222,392	n = 152,987	n = 69,405	
Height loss, cm	0.2 ± 0.7	− 0.1 ± 0.4	0.9 ± 0.5	< 0.001
Age, year	63.4 ± 7.3	63.0 ± 7.5	64.3 ± 6.8	< 0.001
Men	40	41	36	< 0.001
Height, cm	157.1 ± 8.4	157.3 ± 8.4	156.6 ± 8.4	< 0.001
Weight, kg	57.2 ± 10.2	57.4 ± 10.2	56.9 ± 10.1	< 0.001
Body mass index, kg/m^2^	22.9 (20.9–25.0)	22.9 (20.9–25.0)	22.9 (20.9–25.0)	0.827
Waist, cm	83.7 ± 9.0	83.6 ± 8.9	84.0 ± 9.1	< 0.001
Smoke	12	13	11	< 0.001
Hypertension	44	43	45	< 0.001
Diabetes	9	9	10	0.138
Dyslipidemia	43	43	43	0.001
History of stroke	3	3	3	< 0.001
History of cardiovascular disease	6	5	6	< 0.001
Systolic blood pressure, mmHg	128.4 ± 16.9	128.2 ± 16.9	129.0 ± 16.9	< 0.001
Diastolic blood pressure, mmHg	76.2 ± 10.5	76.2 ± 10.5	76.2 ± 10.5	0.858
Fasting blood glucose, mg/dL	96.8 ± 18.4	96.9 ± 18.5	96.6 ± 18.2	< 0.001
HbA1c, %	5.3 ± 0.6	5.3 ± 0.6	5.3 ± 0.6	0.005
Triglyceride, mg/dL	119.5 ± 77.8	120.1 ± 79.1	118.2 ± 74.9	0.003
HDL cholesterol, mg/dL	62.2 ± 16.0	62.1 ± 16.0	62.2 ± 16.0	0.195
LDL cholesterol, mg/dL	126.0 ± 29.9	126.1 ± 29.9	125.9 ± 29.9	0.322

Values are expressed as mean ± standard deviation, medians (interquartile range), or percentage as appropriate.

*BP* blood pressure, *HbA1c* hemoglobin A1c, *HDL* high-density lipoprotein, *LDL* low-density lipoprotein.

**Table 2:** Baseline (2008) characteristics of men stratified into 2-year height loss < 0.5 cm and ≥ 0.5 cm.

	Total	Height loss		P value
		< 0.5 cm	≥ 0.5 cm	
	n = 88,285	n = 63,273	n = 25,012	
Height loss, cm	0.2 ± 0.6	− 0.1 ± 0.4	0.9 ± 0.5	< 0.001
Age, year	63.4 ± 7.7	63.2 ± 7.8	64.0 ± 7.3	< 0.001
Height, cm	164.6 ± 6.1	164.5 ± 6.1	164.7 ± 6.2	< 0.001
Weight, kg	64.3 ± 9.1	64.3 ± 9.1	64.1 ± 9.2	0.011
Body mass index, kg/m^2^	23.5 (21.8–25.4)	23.6 (21.8–25.4)	23.4 (21.7–25.3)	< 0.001
Waist, cm	85.2 ± 7.9	85.2 ± 7.9	85.3 ± 8.1	0.601
Smoke	24	24	24	0.330
Hypertension	49	49	50	0.010
Diabetes	13	13	13	0.410
Dyslipidemia	33	33	32	< 0.001
History of stroke	4	4	5	0.010
History of cardiovascular disease	7	7	8	0.001
Systolic blood pressure, mmHg	130.4 ± 16.6	130.3 ± 16.5	130.6 ± 16.6	0.010
Diastolic blood pressure, mmHg	78.4 ± 10.4	78.4 ± 10.4	78.3 ± 10.5	0.093
Fasting blood glucose, mg/dL	100.9 ± 21.4	101.0 ± 21.5	100.7 ± 21.0	0.020
HbA1c, %	5.4 ± 0.7	5.4 ± 0.7	5.4 ± 0.7	0.949
Triglyceride, mg/dL	134.0 ± 94.8	134.6 ± 95.5	132.5 ± 93.0	< 0.001
HDL cholesterol, mg/dL	57.3 ± 15.1	57.3 ± 15.0	57.4 ± 15.3	0.989
LDL cholesterol, mg/dL	120.5 ± 29.4	120.9 ± 29.4	119.6 ± 29.4	< 0.001

Values are expressed as mean ± standard deviation, medians (interquartile range), or percentage as appropriate.

*BP* blood pressure, *HbA1c* hemoglobin A1c, *HDL* high-density lipoprotein, *LDL* low-density lipoprotein.

**Table 3:** Baseline (2008) characteristics of women stratified into 2-year height loss < 0.5 cm and ≥ 0.5 cm.

	Total	Height loss		P value
		< 0.5 cm	≥ 0.5 cm	
	n = 134,107	n = 89,714	n = 44,393	
Height loss, cm	0.3 ± 0.7	− 0.1 ± 0.5	0.9 ± 0.5	< 0.001
Age, year	63.4 ± 7.0	62.8 ± 7.3	64.4 ± 6.4	< 0.001
Height, cm	152.1 ± 5.5	152.1 ± 5.5	151.4 ± 5.9	< 0.001
Weight, kg	52.6 ± 8.0	52.6 ± 8.0	53.0 ± 8.9	< 0.001
Body mass index, kg/m^2^	22.4 (20.4–24.6)	22.4 (20.4–24.5)	22.7 (20.6–25.1)	< 0.001
Waist, cm	82.7 ± 9.4	82.7 ± 9.4	84.2 ± 10.2	< 0.001
Smoke	4	4	4	< 0.001
Hypertension	40	40	47	< 0.001
Diabetes	7	7	8	< 0.001
Dyslipidemia	50	50	49	0.053
History of stroke	2	2	3	< 0.001
History of cardiovascular disease	5	4	6	< 0.001
Systolic blood pressure, mmHg	127.2 ± 17.1	127.1 ± 17.1	129.7 ± 16.8	< 0.001
Diastolic blood pressure, mmHg	74.8 ± 10.3	74.8 ± 10.3	75.5 ± 10.2	< 0.001
Fasting blood glucose, mg/dL	94.1 ± 15.6	94.1 ± 15.4	94.8 ± 18.1	0.903
HbA1c, %	5.3 ± 0.5	5.3 ± 0.5	5.3 ± 0.6	< 0.001
Triglyceride, mg/dL	110.0 ± 62.5	109.9 ± 62.6	111.0 ± 60.8	< 0.001
HDL cholesterol, mg/dL	65.4 ± 15.8	65.4 ± 15.8	64.0 ± 15.5	< 0.001
LDL cholesterol, mg/dL	129.7 ± 29.7	129.7 ± 29.7	128.8 ± 29.3	0.224

Values are expressed as mean ± standard deviation, medians (interquartile range), or percentage as appropriate. *BP* blood pressure, *HbA1c* hemoglobin A1c, *HDL* high-density lipoprotein, *LDL* low-density lipoprotein.

### Height loss and mortality

During the observation period (mean 4.8 ± 1.1 years), 1436 people (889 men and 547 women) died. The causes of death coded according to the ICD-10 were: neoplasms (n = 773), diseases of the circulatory system (n = 282), injury, poisoning and various other external causes (n = 169), diseases of the respiratory system (n = 66), diseases of the digestive system (n = 41), various infectious and parasitic diseases (n = 29), diseases of the nervous system (n = 21), diseases of the blood and blood-forming organs and various disorders involving the immune mechanism (n = 16), diseases of the musculoskeletal system and connective tissue (n = 15), and others (n = 24). Detailed causes of death and their corresponding ICD-10 codes are shown in the supplemental table (Table S3).

**Figure 2:**
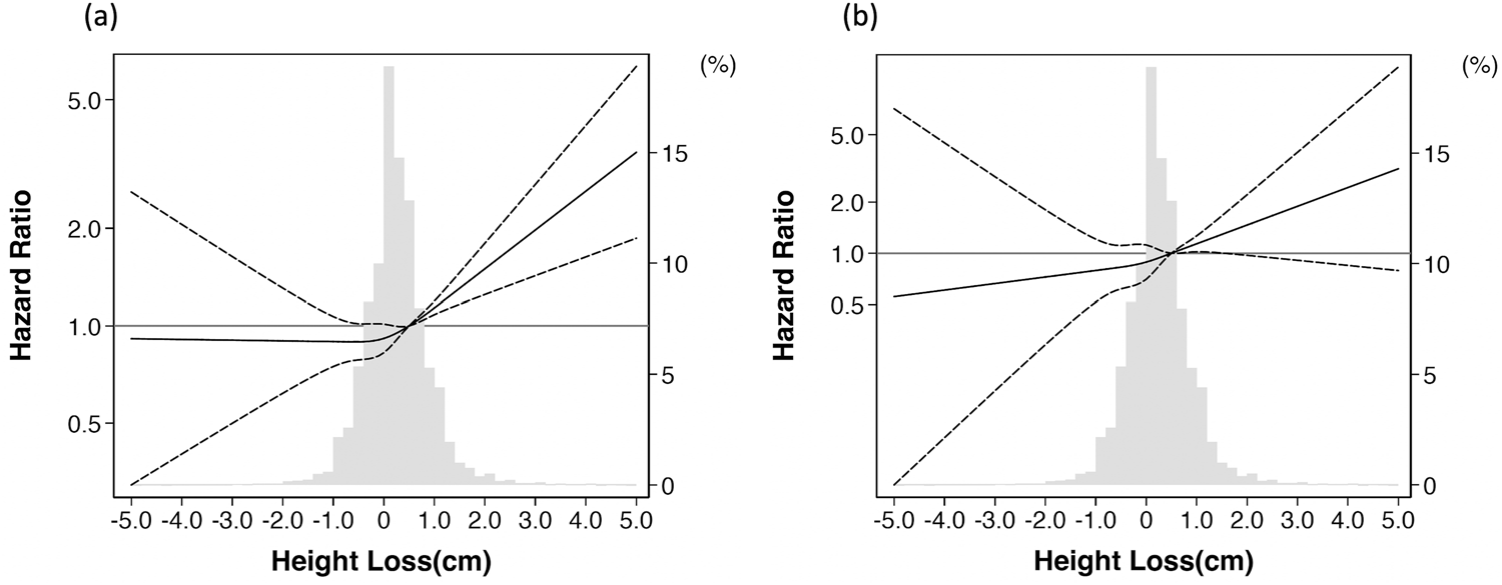
Hazard ratios of height loss of > 0.5 cm over 2 years for overall mortality and cardiovascular mortality. The exposure of interest in this study was height loss over 2 years. Height loss was calculated as baseline (2008) height minus height at follow-up (2010), with a positive value indicating height loss. The vertical axis shows the hazard ratio (versus a reference height loss of 0.5 cm) and 95% confidence interval, assessed using the Cox proportional hazards model, for overall mortality (**a**) and cardiovascular mortality (**b**) until 2014, and the horizontal axis shows the height loss. The mean observation period was 4.8 ± 1.1 years. Solid lines represent the hazard ratio and dotted lines represent the 95% confidence interval of the hazard ratio.

In an unadjusted model, exposure to height loss ≥ 0.5 cm was associated with an increased risk of all-cause mortality compared to height loss < 0.5 cm as the reference. These associations remained significant after additional adjustments in Model 1 and Model 2. The adjusted hazard ratios in Model 2 were 1.26 (95% confidence interval: 1.13–1.41) for exposure to height loss ≥ 0.5 cm compared to height loss < 0.5 cm (Table 4). Using restricted cubic spline functions, increasing levels of height loss were associated with a higher risk of all-cause mortality (Fig. 2a). In unadjusted models, exposure to height loss ≥ 0.5 cm showed significantly higher hazard ratios compared to height loss < 0.5 cm in both men and women. These associations remained significant after adjustments in Model 1 and Model 2.

**Table 4:** Hazard ratios and 95% confidence intervals for the association between height loss and mortality.

Height loss	Number of events	Incident rate ratio (/1000 person-year)	Unadjusted hazard ratios	Adjusted hazard ratios	
				Model 1	Model 2
All-cause mortality					
Total					
< 0.5 cm	932	1.26 (1.18–1.35)		*Reference*	
≥ 0.5 cm	504	1.53 (1.40–1.67)	1.27 (1.14–1.42)	1.27 (1.14–1.42)	1.26 (1.13–1.41)
Men					
< 0.5 cm	599	1.97 (1.82–2.13)		*Reference*	
≥ 0.5 cm	290	2.44 (2.17–2.74)	1.29 (1.12–1.48)	1.25 (1.08–1.44)	1.24 (1.08–1.43)
Women					
< 0.5 cm	333	0.77 (0.69–0.85)		*Reference*	
≥ 0.5 cm	214	1.02 (0.89–1.16)	1.42 (1.19–1.69)	1.29 (1.08–1.54)	1.28 (1.07–1.52)
Cardiovascular mortality					
Total					
< 0.5 cm	179	0.24 (0.21–0.28)		*Reference*	
≥ 0.5 cm	100	0.30 (0.25–0.37)	1.35 (1.05–1.72)	1.35 (1.05–1.73)	1.34 (1.04–1.72)
Men					
< 0.5 cm	118	0.39 (0.32–0.46)		*Reference*	
≥ 0.5 cm	54	0.45 (0.35–0.59)	1.22 (0.88–1.69)	1.19 (0.86–1.65)	1.18 (0.85–1.64)
Women					
< 0.5 cm	61	0.14 (0.11–0.18)		*Reference*	
≥ 0.5 cm	46	0.22 (0.14–0.20)	1.77 (1.20–2.62)	1.61 (1.09–2.39)	1.60 (1.08–2.37)

Model 1 is adjusted for age, sex, and basement height. Model 2 is adjusted for age, sex, baseline height, body mass index, hypertension, diabetes, dyslipidemia, history of stroke, history of cardiovascular disease, and current smoking.

Cardiovascular deaths occurred in only 279 people during the follow-up observation period. In both unadjusted and multivariate-adjusted models, exposure to height loss ≥ 0.5 cm was associated with a greater risk of cardiovascular mortality versus height loss < 0.5 cm as the reference. These significant associations were observed in women, but not in men. Using restricted cubic spline functions, increasing levels of height loss were associated with a higher risk of cardiovascular mortality (Fig. 2b).

Further analysis using other cut-off values of height loss of 1.0 cm and 1.5 cm over 2 years showed similar associations as the above results (Tables S1, S2; Figures S1, S2).

## Discussion

In this observational study of the general Japanese population who underwent specific health check-ups, we found a significant relationship between height loss and all-cause mortality. Significant associations were also found between height loss and cardiovascular mortality in the overall population and in women. In men, on the other hand, although height loss showed an increasing trend for cardiovascular mortality, the associations were not significant. We also found that even a small decrease in height over a 2-year period (height loss of ≥ 0.5 cm) is associated with a risk of all-cause mortality in both men and women.

Associations between height loss and mortality have been shown in several previous studies^7–11^. In those studies, height loss of 2–5 cm or more in the long term (4–20 years) were reported to be related to mortality risk in the elderly^7,8,10,11^ and in northern European women^9^. Although these previous studies had longer observation periods, ranging from several years to a dozen years, the number of participants was not necessarily sufficient. On the other hand, since data on the height changes before baseline measurements were not available in the present study, close observation of height changes for a longer period might be needed to clarify the relationship between height loss and mortality risk, although the present, large-scale study provides evidence that even relatively smaller decreases in height in the short term are associated with an increased risk of mortality in the general population.

An association between height loss and cardiovascular disease has been previously reported^9–11^. In this study, we investigated the relationship between height loss and cardiovascular mortality as a secondary outcome, but we could not find an association between height loss and cardiovascular mortality in men. A recent observational study conducted in South Korea revealed that people with a height loss of greater than 2% had a greater risk of cardiovascular diseases^13^, although subgroup analysis showed significant differences only in the risk of ischemic stroke, but not in myocardial infarction. The limited number of cardiovascular deaths (only 279 in total, 172 in men) in our cohort could be one of the reasons why we found no significant association between height loss and cardiovascular risk in men.

The mechanism by which height loss increases mortality is still unclear. Height loss is mainly caused by vertebral fractures^3^, disc reduction^2^, postural change, and kyphosis^1,25,26^. Vertebral fractures are known to worsen life prognosis^27,28^. Fractures associated with osteoporosis are reportedly associated with a height loss of ≥ 6 cm^29,30^. In addition, hyper-kyphosis is associated with restrictive pulmonary disease^31^, decreased physical function^32–34^, and increased overall mortality^27,35,36^, which suggest that hyper-kyphosis could be a related factor in both height loss and mortality risk. However, the impact of osteoporosis or hyper-kyphosis in the present study might have been limited, since the height loss related to an increased risk of mortality in this study was relatively small (height loss of only ≥ 0.5 cm). A previous study found that height loss was related to overall mortality independent of bone mineral density and vertebral fractures during height loss^7,8^. Loss of skeletal muscle mass (sarcopenia) due to muscle weakness and aging has also been reported as a predictor of mortality^37–39^, and Wannamethee et al. found that sarcopenia was associated with bone loss and poor bone structure in men, which might result in height loss^11^. However, since it is still unclear whether height loss is related to an increased risk of mortality through its association with sarcopenia, further study is still needed to clarify the exact mechanism by which height loss increases the risk of mortality.

Measurement of height is low-cost and straightforward. Our results suggest that 2-year height loss could be one of the useful prognostic factors for mortality risk. Detection of height loss might serve as an impetus for screening for osteoporosis, vertebral fractures and kyphosis. In addition to the above, even a small decrease in height might indicate the future potential risks of skeletal muscle mass loss, sarcopenia, frailty and an increased mortality risk. Therefore, it should be highlighted that height loss might be an important biomarker that reflects not only bone disorders such as osteoporosis, vertebral fracture and kyphosis, but might also be a feature of impaired physical resources, the etiology of sarcopenia and mortality risk, although it has not been recognized as a criterion in the definition of either sarcopenia or frailty.

Only a few studies have evaluated possible interventions to prevent height loss. Alendronate, a drug used in the treatment of osteoporosis, reportedly reduces height loss by improving bone mineral density^40^. Physical activity has also been reported as being protective against height loss in post-menopausal women^41^, and, in a recent study, regular physical exercise reportedly contributed to prevention of height loss in women^9^. Thus, physical exercise might be one of the possible tools to preventing height loss by maintaining skeletal muscle mass, but whether physical exercise or activity prevents not only height loss, but also its associated comorbidities and mortality, should be examined in the future.

A major strength of the present study is that it was a large-scale observational study with participants from all over Japan. However, we should acknowledge several limitations of the present study. First, data on histories of bone disorders (e.g., osteoporosis, vertebral fracture, and kyphosis) before recruitment, data on bone mineral density and the presence or absence of fragility fractures during the observation period, and data on medication use were unavailable since we used data from health check-ups, which might have been a confounding factor. Second, we could not evaluate the causal relationship between height loss and all-cause mortality due to the observational nature of the analyses, and this requires further investigation. Third, there might have been a selection bias because participants who undergo annual health check-ups are considered a relatively health-conscious population. Furthermore, since the participants were younger in the present study (mean age of 63.4 years at baseline), the observation period (mean observational period of 4.8 years) might have been insufficient to provide significant evidence of an association between height loss and mortality. Fourth, as histories of cardiovascular disease and stroke were obtained via patient questionnaires, these data might have included inaccurate diagnosis, although cardiovascular history could be one of the important confounding factors for all-cause and cardiovascular mortality. Fifth, height was measured using a standardized stadiometer by trained staff at specific health checkups, although its measurement sensitivity was not investigated in the present study. Therefore, the calculated height loss might not have been completely accurate due to possible measurement errors, although unreliable height data were excluded from the analysis.

In conclusion, 2-year height loss was associated with an increased risk of mortality among a Japanese nationwide population that underwent health specific check-ups. Our results indicate that even a small decrease in height over a short time period might be a useful marker for stratifying mortality risk. These findings suggest the necessity for more attention to height loss to identify individuals at increased mortality risk. Further research is still needed to clarify the detailed mechanism by which height loss increases mortality risk, and to examine how to prevent height loss and whether prevention of height loss might reduce the mortality risk.

## Supplementary Information

Supplementary Legends.

Supplementary Figure 1.

Supplementary Figure 2.

Supplementary Tables.

## Data Availability

The data that support the findings of this study are available on request from the corresponding author. The data are not publicly available due to privacy and ethical restrictions.
