# Supplementary material for: Association between height loss and mortality in the general population
Source: Sci Rep. 2023 Mar 3;13:3593. doi: 10.1038/s41598-023-30835-1 (PMC9984491; doi:10.1038/s41598-023-30835-1)
Supplement: Supplementary file 1 — Supplementary Legends. [file 41598_2023_30835_MOESM1_ESM.docx]

**Figure legends**

**Supplemental figures**

**Figure S1.** Hazard ratios of height loss of ≥1.0 cm over 2 years for overall mortality and cardiovascular mortality.

The vertical axis shows the hazard ratio (versus a reference height loss of 1.0 cm) and 95% confidence interval, assessed using the Cox proportional hazards model, for overall mortality (a) and cardiovascular mortality (b) until 2014, and the horizontal axis shows the height loss. Solid lines represent the hazard ratio and dotted lines represent the 95% confidence interval of the hazard ratio.

**Figure S2.** Hazard ratios of height loss of ≥1.5 cm over 2 years for overall mortality and cardiovascular mortality.

The vertical axis shows the hazard ratio (reference height loss of 1.5 cm) and 95% confidence interval, assessed using the Cox proportional hazards model, for overall mortality (a) and cardiovascular mortality (b) until 2014, and the horizontal axis shows the height loss. Solid lines represent the hazard ratio and dotted lines represent the 95% confidence interval of the hazard ratio.
