# Supplementary material for: Association between height loss and mortality in the general population
Source: Sci Rep. 2023 Mar 3;13:3593. doi: 10.1038/s41598-023-30835-1 (PMC9984491; doi:10.1038/s41598-023-30835-1)
Supplement: Supplementary file 4 — Supplementary Tables. [file 41598_2023_30835_MOESM4_ESM.docx]

**Table S1.** Hazard ratios and 95% confidence intervals for the association between height loss (≥1.0 cm) and mortality

|  |  | Number of Events | Incident rate ratio (/1000 person-year) | Unadjusted Hazard Ratios | Adjusted Hazard Ratios | |
| --- | --- | --- | --- | --- | --- | --- |
|  |  |  |  |  | Model 1 | Model 2 |
| All-cause mortality | Height loss |  |  |  |  |  |
| Total | <1.0cm | 1253 | 1.31(1.24-1.38) |  | *Reference* |  |
|  | ≥1.0cm | 183 | 1.67 (1.45-1.93) | 1.36 (1.16-1.59) | 1.35 (1.15-1.58) | 1.33 (1.13-1.55) |
|  |  |  |  |  |  |  |
| Men | <1.0cm | 793 | 2.05 (1.91-2.20) |  | *Reference* |  |
|  | ≥1.0cm | 96 | 2.60 (2.13-3.18) | 1.31 (1.05-1.62) | 1.26 (1.02-1.57) | 1.24 (1.00-1.54) |
|  |  |  |  |  |  |  |
| Women | <1.0cm | 460 | 0.80 (0.73-0.88) |  | *Reference* |  |
|  | ≥1.0cm | 87 | 1.20 (0.97-1.48) | 1.65 (1.30-2.08) | 1.43 (1.13-1.81) | 1.40 (1.11-1.77) |
|  |  |  |  |  |  |  |
| Cardiovascular mortality |  |  |  |  |  |  |
| Total | <1.0cm | 242 | 0.25 (0.22-0.29) |  | *Reference* |  |
|  | ≥1.0cm | 37 | 0.34 (0.24-0.47) | 1.45 (1.03-2.06) | 1.46 (1.03-2.06) | 1.42 (1.00-2.01) |
|  |  |  |  |  |  |  |
| Men | <1.0cm | 158 | 0.41 (0.35-0.48) |  | *Reference* |  |
|  | ≥1.0cm | 14 | 0.38 (0.22-0.64) | 0.99 (0.57-1.70) | 0.96 (0.56-1.66) | 0.94 (0.54-1.63) |
|  |  |  |  |  |  |  |
| Women | <1.0cm | 84 | 0.15 (0.12-0.18) |  | *Reference* |  |
|  | ≥1.0cm | 23 | 0.32 (0.21-0.48) | 2.43 (1.53-3.87) | 2.13 (1.33-3.39) | 2.08 (1.31-3.33) |

*Note*: Model 1 is adjusted for age, sex, and basement height. Model 2 is adjusted for age, sex, baseline height, body mass index, hypertension, diabetes, dyslipidemia, history of stroke, history of cardiovascular disease, and current smoking.

**Table S2.** Hazard ratios and 95% confidence intervals for the association between height loss (≥1.5 cm) and mortality

|  |  | Number of Events | Incident rate ratio (/1000 person-year) | Unadjusted Hazard Ratios | Adjusted Hazard Ratios | |
| --- | --- | --- | --- | --- | --- | --- |
|  |  |  |  |  | Model 1 | Model 2 |
| All-cause mortality | Height loss |  |  |  |  |  |
| Total | <1.5cm | 1357 | 1.31 (1.25-1.39) | *Reference* | | |
|  | ≥1.5cm | 79 | 2.23 (1.79-2.78) | 1.86 (1.48-2.34) | 1.81 (1.44-2.28) | 1.77 (1.41-2.23) |
|  |  |  |  |  |  |  |
| Men | <1.5cm | 846 | 2.05 (1.92-2.20) | *Reference* | | |
|  | ≥1.5cm | 43 | 3.83 (2.84-5.16) | 1.97 (1.45-2.69) | 1.86 (1.36-2.54) | 1.82 (1.33-2.48) |
|  |  |  |  |  |  |  |
| Women | <1.5cm | 511 | 0.82 (0.75-0.90) | *Reference* | | |
|  | ≥1.5cm | 36 | 1.49 (1.07-2.06) | 2.07 (1.48-2.91) | 1.72 (1.22-2.41) | 1.68 (1.19-2.35) |
|  |  |  |  |  |  |  |
| Cardiovascular mortality |  |  |  |  |  |  |
| Total | <1.5cm | 261 | 0.25 (0.22-0.29) | *Reference* | | |
|  | ≥1.5cm | 18 | 0.51 (0.32-0.81) | 2.21 (1.37-3.56) | 2.18 (1.35-3.52) | 2.10 (1.30-3.39) |
|  |  |  |  |  |  |  |
| Men | <1.5cm | 166 | 0.40 (0.35-0.47) | *Reference* | | |
|  | ≥1.5cm | 6 | 0.53 (0.34-1.19) | 1.42 (0.63-3.21) | 1.36 (0.60-3.08) | 1.31 (0.58-2.95) |
|  |  |  |  |  |  |  |
| Women | <1.5cm | 95 | 0.15 (0.13-0.19) | *Reference* | | |
|  | ≥1.5cm | 12 | 0.50 (0.28-0.87) | 3.69 (2.02-6.73) | 3.07 (1.68-5.64) | 2.99 (1.63-5.48) |

*Note*: Model 1 is adjusted for age, sex, and basement height. Model 2 is adjusted for age, sex, baseline height, body mass index, hypertension, diabetes, dyslipidemia, history of stroke, history of cardiovascular disease, and current smoking.

**Table S3.** Causes of death in the study population and their corresponding ICD-10 codes

| The causes of death | ICD-10 codes | N | % |
| --- | --- | --- | --- |
| Neoplasms | C00-D48 | 773 | 53.8 |
| Diseases of the circulatory system | I00-I99 | 282 | 19.6 |
| Injury, poisoning and certain other consequences of external causes | S00-T98 | 169 | 11.8 |
| Diseases of the respiratory system | J00-J99 | 66 | 4.6 |
| Diseases of the digestive system | K00-K93 | 41 | 2.9 |
| Certain infectious and parasitic diseases | A00-B99 | 29 | 2.0 |
| Diseases of the nervous system | G00-G99 | 21 | 1.5 |
| Diseases of the blood and blood-forming organs and certain disorders involving the immune mechanism | R00-R99 | 16 | 1.1 |
| Diseases of the musculoskeletal system and connective tissue | M00-M99 | 15 | 1.0 |
| Diseases of the genitourinary system | N00-N99 | 6 | 0.4 |
| Diseases of the blood and blood-forming organs and certain disorders involving the immune mechanism | D50-D89 | 5 | 0.3 |
| Endocrine, nutritional, and metabolic diseases | E00-E90 | 5 | 0.3 |
| Diseases of the skin and subcutaneous tissue | L00-L99 | 1 | 0.1 |
| Unknown | N/A | 7 | 0.5 |

*Note*: Abbreviations: ICD-10, International Classification of Diseases, 10^th^ revision, N/A, not applicable.
